# Impaired Pre‐Critical Illness Quality of Life in Elderly ICU Patients

**DOI:** 10.1111/aas.70304

**Published:** 2026-07-26

**Authors:** Shirin K. Frisvold, Bjørn A. Kroken, Daniel Bergum, Kristin Sandal‐Berg, Ole K. Fossum, Pål Klepstad, Reidar Kvåle, Hans Flaatten, Andrew M. Garratt

**Affiliations:** ^1^ Department of Clinical Medicine UiT the Arctic University of Norway Tromso Norway; ^2^ Department of Anaesthesia and Intensive Care University Hospital of North Norway Tromso Norway; ^3^ Department of Anaesthesiology and Intensive Care Medicine St Olavs University Hospital Trondheim Norway; ^4^ Department of Anaesthesia and Intensive Care Medicine Nordland Hospital Trust Bodø Norway; ^5^ Faculty of Nursing and Health Sciences, Nord University Bodø Norway; ^6^ Department of Anaesthesia and Intensive Care Akershus University Hospital Oslo Norway; ^7^ Department of Clinical Medicine University of Bergen Bergen Norway; ^8^ Department of Anaesthesia and Intensive Care Haukeland University Hospital Bergen Norway; ^9^ Department of Research and Development Haukeland University Hospital Bergen Norway; ^10^ Division for the Health Services Norwegian Institute of Public Health Oslo Norway

## Abstract

**Background:**

The EuroQol EQ‐5D‐5L is recommended to assess health‐related quality of life (HRQoL) in intensive care unit (ICU) survivors and included in many national ICU registries at the time of follow‐up. While establishing baseline HRQoL in elderly ICU survivors is important for informing follow‐up services, it is rarely performed in clinical practice. We aimed to compare pre‐critical illness EQ‐5D‐5L scores with Norwegian general population norms in elderly adults.

**Methods:**

We conducted a prospective multicenter observational study across five ICUs to assess pre‐critical illness HRQoL in mechanically ventilated patients ≥ 65 years old using EQ‐5D‐5L. Data assessing HRQoL before critical illness were collected during the ICU stay, obtained primarily from proxies or via patient self‐report where feasible. Response frequencies for the EQ‐5D‐5L dimensions, EQ‐5D‐5L index, and EQ VAS scores were compared with those for general population norms after random exact matching for age and sex. The five dimensions were dichotomized into the presence of health problems or not.

**Results:**

Based on responses from 345 participants, the ICU population had a baseline mean EQ‐5D‐5L index of 0.78 (±0.24), an EQ VAS of 61 (±23), and 60 ICU patients reported no problems in any of the five domains. The norm population had a mean EQ‐5D‐5L index of 0.88 (±0.17), an EQ VAS of 79 (±20), and 107 had no problems in any of the five dimensions. Pearson chi‐square or two‐tailed *t*‐tests showed significantly (*p* < 0.05) poorer scores for all EQ‐5D‐5L scores for the ICU population compared to the norm data. The greatest differences were found in the younger age group 65–72 years. Comorbidity was a strong independent predictor of baseline health status, whereas chronological age showed no significant association.

**Conclusion:**

Compared to the age‐ and sex‐matched general population, the elderly ICU population had poorer HRQoL before critical illness occurred. To improve the interpretation of post‐ICU assessments, baseline EQ‐5D‐5L should be assessed at ICU admission. The results highlight the need for studies in ICU patients that include HRQoL as part of a prospective study design.

**Editorial Comment:**

This study presents pre‐ICU quality of life scoring for older cases requiring mechanical ventilation. Compared to a matched non‐ICU cohort, significant lower reported quality of life scoring was observed.

## 1 Introduction

Mortality is a well‐established endpoint in intensive care research, but outcomes that matter to survivors, including health‐related quality of life (HRQoL), receive far less attention despite their relevance for clinical and cost‐effectiveness studies [1, 2]. These outcomes are assessed by means of standardized questionnaires, often referred to as patient‐reported outcome measures.

The EQ‐5D‐5L is a generic patient‐reported outcome measure that is widely used across health problems and general population norm data is available to aid interpretation [3, 4, 5]. It has long been recommended for ICU studies and economic evaluation [3, 6, 7, 8]. Because many ICU patients are too ill to self‐complete, proxy completion by health personnel or next of kin may be necessary and reliable [9]. The EQ‐5D has been included in several studies of elderly ICU patients [10, 11, 12, 13, 14, 15] and a meta‐analysis concluded that EQ‐5D should inform patient‐centered decision making in elderly patients [16]. Baseline EQ‐5D combined with geriatric assessment predicted mortality [10], and EQ‐5D scores were associated with frailty and recovery [11, 13, 14, 15].

ICU survivors experience substantially reduced HRQoL, which affects comorbidity, path of recovery, and the cost‐effectiveness of care, underlining the importance of risk prediction and stratification [17, 18, 19]. Pre‐ICU HRQoL has been shown to be the most important predictor of long‐term HRQoL [20] and mapping pre‐illness status helps identify the patients that are most likely to benefit from rehabilitation and follow‐up. However, pre‐ICU HRQoL is seldom assessed, leaving a gap in our understanding of pre‐existing health trajectory. Recently, the Norwegian EQ‐5D‐5L value set and norm data became available, which enhances the legitimacy and interpretation of results in Norwegian patient populations [5, 21]. Whether population norms adequately reflect baseline HRQoL in patients who become critically ill is uncertain, and the few studies comparing baseline HRQoL with population norms report contradictory findings [22, 23]. Establishing this baseline relative to population norms will also inform economic evaluation. To our knowledge, pre‐ICU EQ‐5D‐5L scores have not been described or compared with general population norm data.

The primary aim of this study was to compare pre–critical illness (baseline) EQ‐5D‐5L health status in patients ≥ 65 years old requiring intensive care and invasive mechanical ventilation with age‐ and sex‐stratified general population norms, testing the hypothesis that the ICU cohort has lower baseline health status (or pre‐ICU functional ability) than the general population. Secondary aims were to compare dimension‐level problem prevalence, EQ‐5D‐5L index and EQ VAS score distributions, including ceiling and floor proportions and observed ranges. This study provides the first pre‐ICU EQ‐5D‐5L index data from Scandinavia.

## 2 Methods

### 2.1 Data Collection

The ICU study was a multicenter prospective, observational study [24], approved by the Norwegian Regional Committee for Medical and Health Research Ethics (172,784/REK North). A signed informed consent was obtained from every included patient or their next of kin. Patients were recruited from five Norwegian ICU's across the four health regions, between June 2022 and July 2024. All Norwegian hospitals are mixed‐bed ICUs with 5 to 10 beds.

Inclusion criteria were patients aged ≥ 65 years requiring invasive mechanical ventilation for ≥ 24 h. Exclusion criteria were lack of written consent from the patient or next of kin, residency outside of Norway, and inability to complete the questionnaire due to cognitive/physical limitations and the absence of next of kin. Data were collected by trained study staff.

The baseline EQ‐5D‐5L was used to assess HRQoL before critical illness, often set to 2 weeks prior to ICU admission, using proxy completion at time of enrolment in the ICU, during which time the patients were non‐responsive. The caregivers were instructed to rate the patient's HRQoL based on how they believed the patient would respond, using self‐completion (pen and paper) or by interview. Patient self‐report was obtained at the ICU whenever feasible. Additional data included patient characteristics such as age, sex, residence, Comorbidity‐Polypharmacy Score, reason for ICU admission, and Sequential Organ Failure Assessment (SOFA).

The study was registered on ClinicalTrials.gov (ID: NCT06012942). We used the STROBE reporting guideline [25] to draft this manuscript, and the STROBE reporting checklist when editing.

### 2.2 Patient‐Reported Outcomes Measures

The EQ‐5D‐5L comprises five questions or dimensions (mobility, self‐care, usual activities, pain/discomfort, anxiety/depression) with five response levels from no problems to extreme problems/unable to do. Health states arising from the dimensions are transformed to a single index using a scoring algorithm derived from valuation tasks undertaken with general population samples to give a national value set [21]. Norwegian EQ‐5D values range from −0.453 to 1; 1 is the best possible health state, and negative values represent states worse than dead, which is equal to 0 [21]. EQ‐5D‐5L dimension scores can also be dichotomized into “no problem” vs. “any problems” [26]. In addition, the EQ VAS assesses the perceived health status using a visual analog scale, with 0–100 endpoints labeled the worst and best imaginable health state, respectively.

The EQ‐5D has undergone considerable testing in Norwegian populations and is the most widely used patient‐reported instrument in the Norwegian national medical registers [21, 26]. Estimates for the minimal important difference (MID) or the smallest difference between groups that is considered important range from 0.024 to 0.100 for the EQ‐5D index and 5 to 10 points for EQ VAS [5, 27, 28, 29, 30, 31, 32]. Estimates from intensive care cohorts lie at the upper end of these ranges [33]. Norwegian EQ‐5D‐5L general population norm data have been used to aid the interpretation of scores and are based on EQ‐5D‐5L responses from a random selection of the general population aged 18 years and older selected from the National Population Register [26]. In total, 3200 (26%) responded to a postal survey distributed in 2019 [5].

### 2.3 Statistical Analysis

Descriptive statistics are presented with mean ± SD, median (range), or number (%), as appropriate. While EQ‐5D‐5L index and EQ VAS scores are often reported as means (SD), we also present medians (IQR) due to the skewed nature of the data and to facilitate direct comparison with prior critical care studies. Response frequencies for the EQ‐5D‐5L dimensions, EQ‐5D‐5L index, and EQ VAS scores were compared with those for general population norms after random exact matching for age and sex to give the same number of norms as patients. Scores ranges were assessed including the percentages scoring at the floor and ceiling compared to the general population [26].

Differences in dimension scores between general and ICU population were assessed using Pearson chi‐square tests. Monte Carlo simulated *p*‐values were used when expected cell counts were < 5. *p*‐values were adjusted for multiple comparisons using the Benjamini–Hochberg procedure, with statistical significance defined as an adjusted *p*‐value < 0.05 (corresponding to a false positive rate of 5%). Between‐group differences (ICU vs. general population) of EQ‐5D‐5L index and EQ VAS scores were assessed with the two‐sample *t*‐test. Interaction by sex was assessed using linear regression models including a population group × sex interaction term for each EQ‐5D‐5L score. Spearman's rank correlation was used to explore the association between the Comorbidity‐Polypharmacy Score and EQ‐5D‐5L index and EQ VAS scores. To identify if comorbidity was an independent predictor, multivariate linear regression models were constructed for both EQ‐5D‐5L index and EQ VAS, adjusting for age.

The prevalence of EQ‐5D health problems (score > 1) was calculated for each dimension by age group. Confidence intervals (CIs) for the proportions were calculated using the Wilson score method. All tests were two‐sided and *p* < 0.05 was considered statistically significant. Statistical analysis was performed using R, version 2024.09.

## 3 Results

### 3.1 Study Population

**FIGURE 1:**
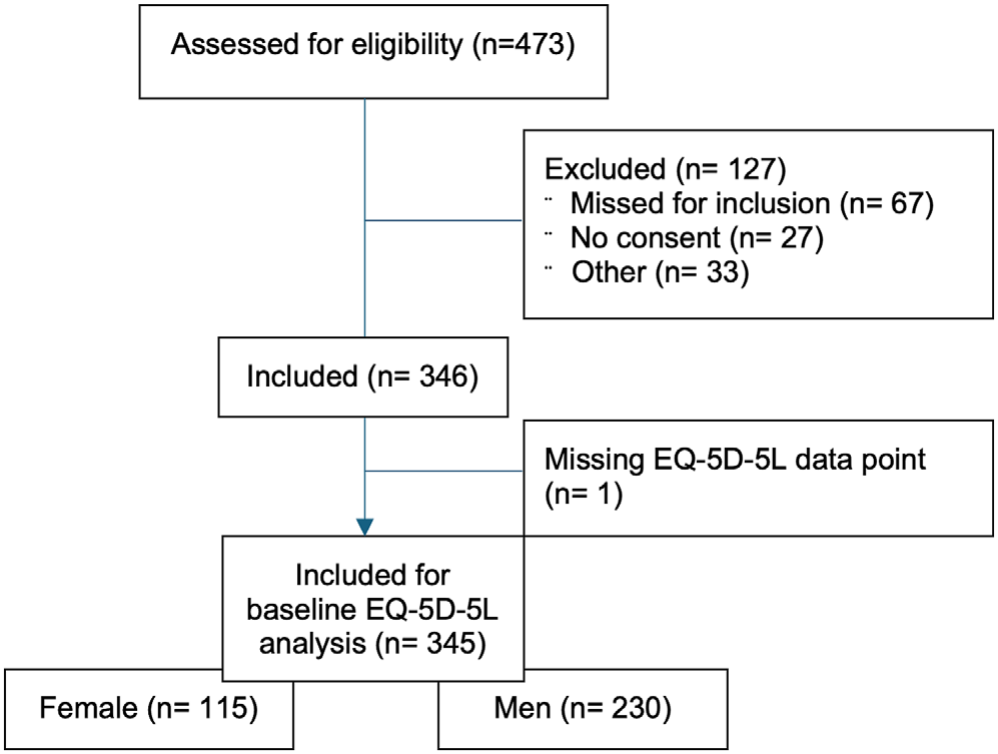
Study flowchart of the ICU cohort.

345 patients from the five ICUs were included in the analysis of the ICU cohort after excluding 128 patients, mainly due to missed inclusion (Figure 1). Initially, 346 patients were included, but one was excluded due to missing EQ‐5D‐5L data. Demographics, clinical, and severity of illness for the ICU population are summarized in Table 1. The mean age was 74 (SD 6), 230 (67%) were male, and 338 (98%) were admitted from home. In order of frequency, the five most common main reasons for admission were sepsis, respiratory failure, CNS disease, emergency surgery, or circulatory failure. The median Comorbidity Polypharmacy Score was 8 (IQR, 5–12).

**TABLE 1:** Characteristics for the intensive care population completing the EQ‐5D‐5L (*n* = 345).

	*N*	%
Age years, mean (SD)	73.9 (5.6)	
Female	115	33.3
Admitted from		
Home	338	98.0
Institution	7	2.0
Admission diagnosis		
Sepsis	68	19.7
Respiratory failure	66	19.1
CNS (non‐trauma)	55	15.9
Emergency surgery	55	15.9
Circulatory failure	42	12.2
Comorbidity Polypharmacy Score, median (IQR)	8 (7)	
Sequential Organ Failure Assessment mean days 1–4, median (IQR)	2.1 (1.1)	

### 3.2 EQ‐5D‐5L Scores

There were significant differences between the ICU population before critical illness and the norms across the five dimensions of EQ‐5D‐5L (Table 2). The first column in Table 2 shows that compared to the age and sex matched general population, ICU patients reported between 6% (pain/discomfort) and 28% (mobility) more problems across EQ‐5D‐5L dimensions, with fewer scoring at the floor. While more patients scored at the ceiling across dimensions, this was still below 3%. The mean EQ‐5D‐5L index score for the ICU population was 0.78 (SD 0.24) compared to 0.88 (SD 0.17) for the norms which was significant (Table 2). The mean EQ VAS score for the ICU population was 61 (SD 23) compared to 78 (SD 20) for the norms which was significant. Both differences exceed or are in the upper range of estimates for MID. Moreover, compared to the general population, approximately half as many patients had the highest possible scores denoting best possible health (EQ‐5D‐5L index 60 vs. 109; EQ VAS scores 10 vs. 21). The patients had both greater score ranges and numbers of health states assessed by the index and EQ VAS.

**TABLE 2:** EQ‐5D‐5L frequencies (%) and descriptive statistics for the general population and intensive care patients (*n* = 345 per cohort).

EQ‐5D dimension/scores, *n* (%)	No problems	Slight problems	Moderate problems	Severe problems	Unable/extreme	Sign.
General population						
Mobility	255 (73.9)	55 (15.9)	19 (5.5)	14 (4.1)	2 (0.6)	
Self‐care	312 (90.4)	27 (7.8)	3 (0.9)	2 (0.6)	1 (0.3)	
Usual activities	261 (75.7)	55 (15.9)	16 (4.6)	9 (2.6)	4 (1.2)	
Pain/discomfort	120 (34.8)	158 (45.8)	39 (11.3)	23 (6.7)	5 (1.4)	
Anxiety/depression	261 (75.7)	60 (17.4)	22 (6.4)	2 (0.6)	0 (0.0)	
Intensive care						
Mobility	160 (46.4)	83 (24.1)	58 (16.8)	37 (10.7)	7 (2.0)	**
Self‐care	282 (81.7)	30 (8.7)	18 (5.2)	11 (3.2)	4 (1.2)	**
Usual activities	202 (58.6)	62 (18.0)	43 (12.5)	28 (8.1)	10 (2.9)	**
Pain/discomfort	101 (29.4)	137 (39.8)	66 (19.2)	31 (9.0)	9 (2.6)	*
Anxiety/depression	194 (56.4)	87 (25.3)	50 (14.5)	12 (3.5)	1 (0.3)	**

	Mean (SD)	Median (IQR)	Best possible	Worst possible	Number of health states	Range
General population						
EQ‐5D‐5L index	0.875 (0.168)	0.941 (0.151)	107 (31.0)	0 (0.0)	73	−0.067‐1
EQ VAS	78.4 (20.3)	85 (20)	29 (8.4)	1 (0.3)	29	0–100
Intensive care						
EQ‐5D‐5L index	0.779 (0.244)**	0.869 (0.245)	60 (17.4)	0 (0.0)	131	−0.320‐1
EQ VAS	61.1 (23.1)**	65 (35)	10 (3.0)	1 (0.3)	45	0–100

*Note:* Statistically significant differences found for all EQ‐5D‐5L dimensions, index and EQ VAS scores in comparisons with general population. Reported *p*‐values for dimensions are adjusted for multiple comparisons using the Benjamini–Hochberg procedure. The number of responses in the ICU cohort for EQ VAS was 333. For the dimensions/index there were 344 or 345 responses.

* *p* < 0.05.

** *p* < 0.01.

There were 306 proxy and 39 patient responses which are shown for the dimensions in Supporting Information, Table S1A,B. Given the difference in sample size and the descriptive nature of this subgroup comparison, formal statistical testing between patients and proxies was not performed to avoid underpowered conclusions.

In the ICU patients, a significant difference between men and women was found for the anxiety/depression dimension, with no differences in the other EQ‐5D‐5L scores (Supporting Information, Table S2). No statistically significant sex differences were found for the norms. Consequently, results for each population are presented with sexes pooled and stratified only by age group. Interaction analyses showed population‐by‐sex interactions for only pain/discomfort and anxiety/depression, indicating that the differences between the ICU patients and norms varied by sex for only these dimensions. No significant interactions were observed for mobility, self‐care, usual activities, EQ‐VAS, or the EQ‐5D index, suggesting that population differences for these outcomes were similar in men and women. Given that no interactions were observed for three dimensions, EQ‐5D index or EQ‐VAS, the datasets were pooled for subsequent analyses.

**FIGURE 2:**
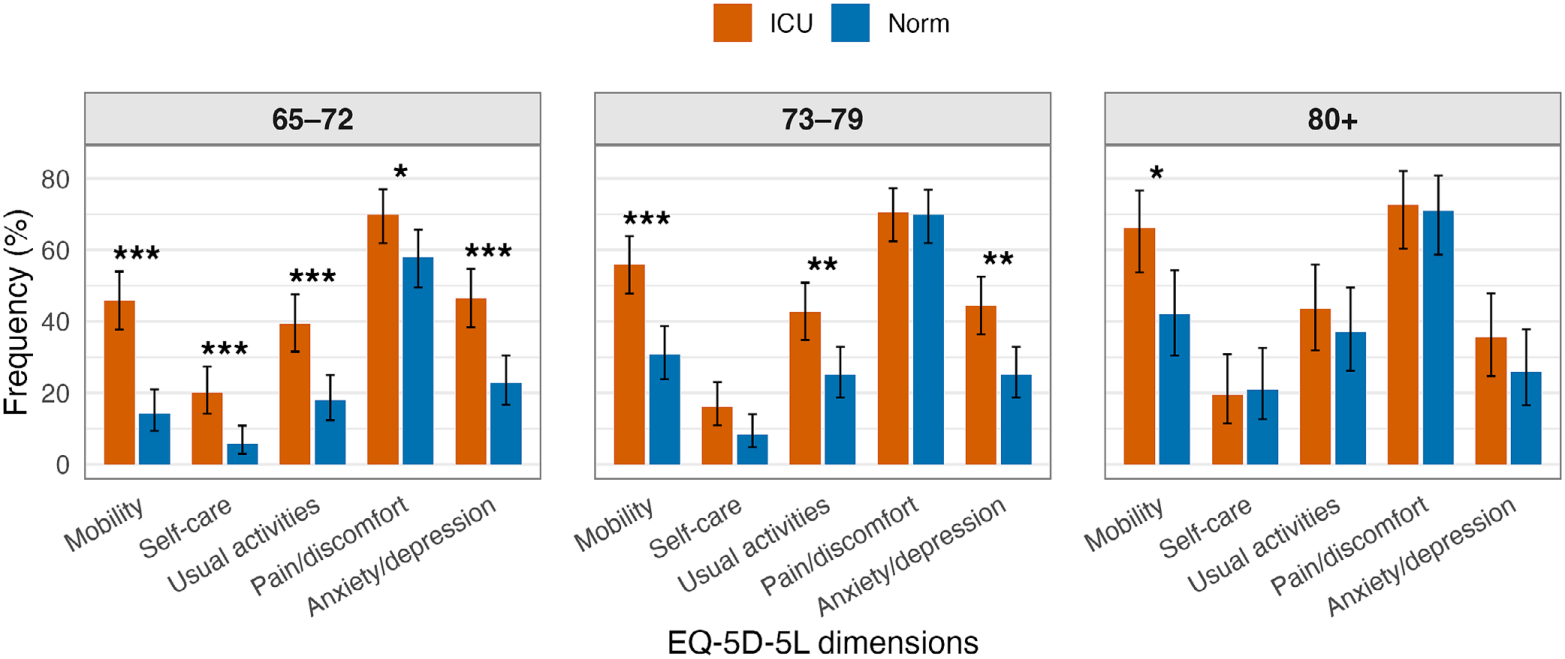
Prevalence of EQ‐5D‐5L health problems (score > 1) by dimension and age group. Bars represent the percentage of participants reporting problems in each dimension for the intensive‐care and the norm cohorts. Error bars indicate 95% confidence intervals; *p*‐value thresholds are shown with stars (*** < 0.001, ** < 0.01, * < 0.05).

There were 109 (31.0%, CI: 26.4%–36.1%) of the norms with no problems in any of the five dimensions compared to 60 (17.4%, CI: 13.8%–21.8%) of the ICU patients. Figure 2 and Supporting Information, Table S3A,B show the percentage of ICU patients and norms reporting problems by dimension for the three age categories. For those 65–72 years old, ICU patients reported more problems with all dimensions. For those of 73–79 years old, significantly more reported problems with mobility, usual activities, and anxiety/depression. For the oldest age group, significantly more of the ICU patients had problems with mobility only.

**FIGURE 3:**
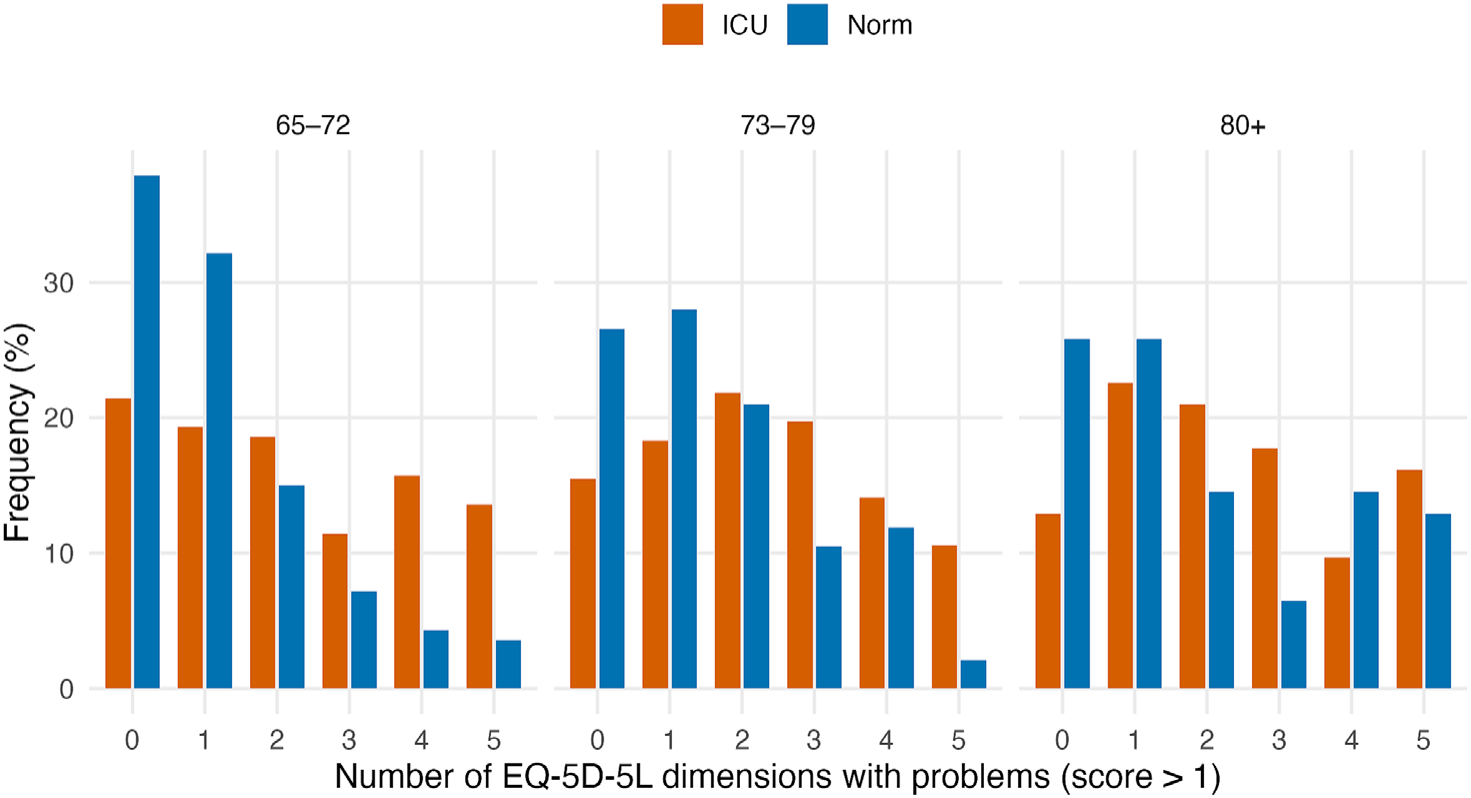
Overall burden of EQ‐5D‐5L problems in the intensive‐care and the norm cohorts. For each age group, the bars show the proportion of patients with 0, 1, 2, 3, 4, or 5 EQ‐5D‐5L dimensions with problems (score > 1), separately for the intensive‐care and the norm cohorts. The distribution of the number of problematic dimensions differed significantly between groups (*χ* ^2^ test, *p* < 0.001).

Figure 3 gives the total number of dimensions (0–5) for which problems were reported by the norms and ICU patients. The differences were significant and were greatest for the youngest age group, both in terms of those reporting no problems and those reporting four or more problems compared to the general population. Since there were population‐by‐sex interactions for two dimensions, we also performed analyses of the individual EQ‐5D‐5L dimensions for each sex (Supporting Information, Table S4A,B and Figure S1A,B). Sex‐stratified analyses largely reflected the pooled data, primarily demonstrating significant differences between the ICU patients and norms within the two youngest age groups. However, a significantly higher proportion of women in the two oldest age groups in the ICU group reported anxiety/depression compared to the norms, a difference absent in men. Men reported significantly more problems than norms in primarily the youngest group.

**FIGURE 4:**
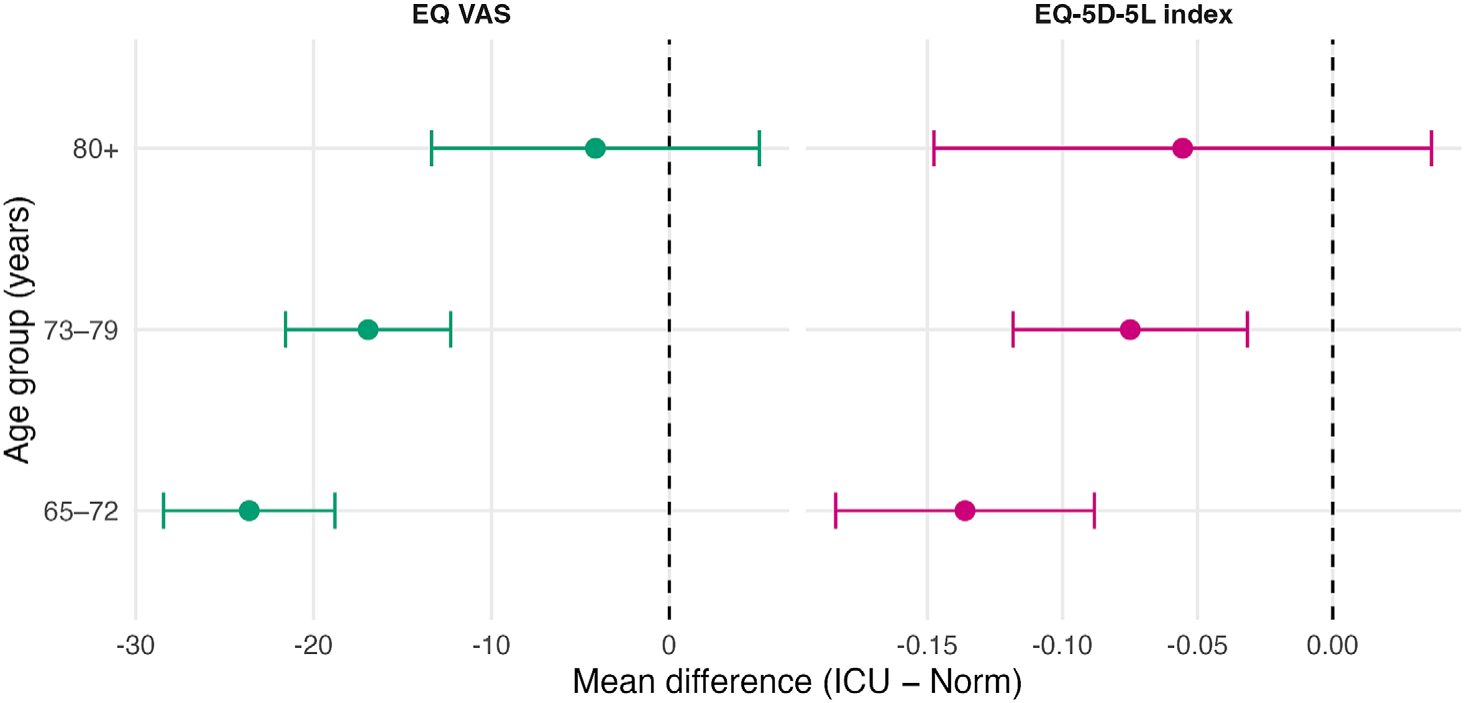
Forest plot showing mean differences (intensive‐care patients–norms) in EQ VAS and EQ‐5D‐5L index by age group, with 95% confidence intervals.

Figure 4 shows forest plots for the EQ‐5D‐5L index and EQ VAS scores for the same age categories. All scores were lower in the ICU patients compared to the norms and differences were greatest in the 65–72 years age group. EQ VAS scores for those of 73–79 years of age were relatively lower when compared to the index scores across the three age groups.

In multivariate linear regression models, the Comorbidity‐Polypharmacy Score was a significant independent predictor of both the ICU baseline EQ‐5D‐5L index scores (*β* = −0.0195; 95% CI, −0.024 to −0.015; *p* < 0.001) and EQ VAS scores (*β* = −1.805; 95% CI, −2.26 to −1.35; *p* < 0.001). After adjusting for comorbidity burden, age was not significantly associated with the baseline index (*p* = 0.69) or the VAS (*p* = 0.09). EQ‐5D‐5L index scores and EQ‐VAS scores plotted against the Comorbidity‐Polypharmacy Score are presented in Supporting Information, Figure S2.

## 4 Discussion

In this Norwegian multicenter study of ICU patients, pre‐critical illness EQ‐5D‐5L scores are poorer than those for age and sex matched general population norm. The magnitude of the difference between the ICU patients and these norms was greatest for the youngest age group 65–72 years and decreased for the two older age groups. Our analysis suggests that this is driven by comorbidity burden rather than age. There were also a greater number and range of EQ‐5D‐5L states for patients, which is evidence that the instrument assesses aspects of HRQoL of relevance to ICU patients. Compared to the norms, floor effects across the dimensions were lower in the patients, and while there were more patients with the worst possible health scoring at the ceiling, these were under 3%. These findings suggest that EQ‐5D‐5L before critical illness should be assessed at admission and that general population data has limitations as a surrogate for baseline scores when performing follow‐up studies of ICU survivors.

The EQ‐5D is recommended as a core outcome measure for critically ill patients and has been assessed for measurement properties in this context [33, 34]. The use of retrospectively collected baseline data following a health event rather than using population norms has been supported by other studies [22, 35]. Pre‐ICU EQ‐5D‐5L in the form of a baseline is rarely measured, which might have implications for generalizability and affect conclusions relating to post‐ICU HRQoL outcomes. This might reflect the difficulties associated with pre‐critical illness retrospective HRQoL measurement, including feasibility and recourse to proxy responses. Few studies have assessed pre‐ICU EQ‐5D, and most present the index or EQ VAS scores. In general, cohorts were younger than ours, and many studies are from more than a decade ago. An older study of elderly patients (mean age 75 years) in a Spanish ICU with high functional status reported a mean EQ VAS of 72 [10]. A more recent Belgian two‐center study reported median EQ VAS of 75–80 in non‐COVID ICU survivors and 85–90 in COVID ICU survivors (median age 66 years) [36]. The baseline scores were compared with 1‐year follow‐up, and the higher scores compared with our cohort might reflect patient selection and ICU case‐mix. In the Australian PREDICT cohort (median age 58 years), the baseline EQ VAS—defined as 1 month before ICU admission and collected at 3 months—had a median 70 [33]. An Argentinian ICU cohort (mean age 60 years) reported baseline medians for the index of 0.80 and EQ VAS of 70 [37]. A retrospective Finnish multicenter registry (mean age 62 years) reported a baseline EQ‐5D index median of 0.68 and EQ VAS 60, obtained during the ICU stay [38]. Another Finnish study of elderly ICU cardiac arrest patients (mean age 66 years) reported a baseline EQ‐5D‐3L median index of 0.89, suggesting baseline HRQoL may vary by ICU diagnosis [39].

Few studies have directly compared baseline pre‐ICU EQ‐5D‐5L with age matched general population norm data, and findings are mixed. In our study, the norms had higher levels of HRQoL compared to the pre‐critical illness population, with the latter showing greater health problems across important health dimensions. These differences were less pronounced in patients aged 80 years and older, although women still reported significantly higher levels of anxiety/depression than their normative counterparts. Assessing pre‐critical illness HRQoL is therefore highly relevant for ICU decision‐making and targeted post‐ICU follow‐up. Conversely, a Finnish single‐centre study in severe trauma patients (age ≥ 65 years) collected baseline EQ‐5D‐3L as soon as possible following admission and found baseline pre‐injury HRQoL exceeded population norms [40]. This might reflect different demographics and/or higher activity levels in a trauma ICU population compared with general ICU cohorts. However, comparisons across all studies are further hampered by variation in the methods of baseline assessment including timing of completion, time frame for retrospective EQ‐5D assessment, and proxy completion.

There is growing recognition of the clinical difference between biological and chronological age [41, 42]. Although the effect of comorbidity on HRQoL or mortality has been documented by some studies [43], the findings are mixed [44, 45]. Our results suggest that the decline in pre‐critical illness HRQoL might be primarily driven by the accumulation of chronic health deficits. Consequently, clinical assessments should consider the evaluation of comorbidity when estimating a patient's baseline health trajectory.

Finally, baseline HRQoL can contribute to a more comprehensive assessment of the clinical‐ and cost‐effectiveness of critical care interventions by isolating the path of decline and recovery attributable to the illness. However, the comparison of post‐ICU HRQoL of survivors with that of the general population is important in terms of assessing the burden of critical illness and informing the goal of recovery for patients.

### 4.1 Strengths and Limitations

Our study has various strengths. First, it uses a multi‐center ICU cohort [24]. Second, the data were collected by dedicated study personnel, which contributed to standardization and near‐complete data. Third, methods of reporting the EQ‐5D‐5L made full use of available data including the five dimensions, index, and EQ VAS scores. Fourth, the EQ‐5D‐5L is a widely used brief generic patient‐reported outcome measure that includes aspects of health of broad importance across health problems. Widespread use means that national scoring exists for the index based on the values of the Norwegian general population, together with norm data, and MID estimates are available to further aid interpretation [5, 21, 26]. Based on a structured search of the literature, this represents the first comparison of pre‐critical illness EQ‐5D‐5L scores with national population norms in a Scandinavian setting of elderly ICU patients.

There are several limitations to our study. The ICU study population was highly selected, consisting exclusively of patients aged ≥ 65 years who required invasive mechanical ventilation for ≥ 24 h. Consequently, our results may not be generalizable to the broader ICU population. Additionally, several patients were eligible but excluded from the study for various reasons. Because detailed demographic and clinical data for excluded patients were unavailable due to data privacy constraints, the potential for bias cannot be entirely excluded. MID estimates for EQ‐5D vary across studies and have been criticized for inconsistent terminology and methodology [26]; nonetheless, the differences observed between pre‐ICU scores and population norms in our cohort were close to or above estimates used in Norwegian and ICU studies [26, 33, 46].

Pre‐critical illness EQ‐5D‐5L was retrospectively assessed, which may be prone to recall bias, response shift or a difficulty in distinguishing a gradual progression of underlying frailty from the acute illness itself [47]. However, our data was assessed close to the time of the critical illness to minimize the potential impact of these factors [48], whereas other studies have assessed baseline HRQL months after ICU discharge [33, 36]. There is evidence supporting the reliability and validity of retrospective EQ‐5D‐5L estimates, including agreement with prospectively collected data, but further evaluation in ICU populations is recommended [49, 50, 51]. This could include test–retest assessments or, resources permitting, prospective pre‐ and post‐care administration to quantify and, if necessary, adjust for response bias.

During the ICU stay, assessments were predominantly proxy‐reported, which may introduce bias compared with self‐report. Proxy reports may underestimate HRQoL due to pessimism bias [23, 52]. Although we report the proxy and patient responses, the study was not designed to assess intra‐individual differences and paired self‐ and proxy‐reports were unavailable. Hence the extent of their agreement could not be assessed. Further research into retrospective measurement and the evaluation of proxy completion in obtaining pre‐critical illness HRQoL data are recommended in these patients as part of prospective population‐based studies.

## 5 Conclusions

Our results showed that EQ‐5D‐5L problems were more frequently reported among the elderly population who became critically ill compared to those of age‐ and sex‐matched general population norms. This difference was probably due to pre‐existing comorbidities and was most pronounced in the youngest age group (65–72 years). In addition, a wide range of health states were captured compared to the general population, showing the EQ‐5D‐5L to be highly relevant for the assessment of the HRQoL in this population. Our study underscores the importance of incorporating pre‐ICU HRQoL data in the interpretation of HRQoL among ICU survivors to enhance understanding of ICU trajectories.

## Author Contributions

S.K.F., A.M.G., B.A.K., and H.F. conceived this study. B.A.K., D.B., K.S.‐B., and O.K.F. collected the ICU cohort data. A.M.G. and S.K.F. were responsible for the design and conduct of the statistical analysis. A.M.G. and S.K.F. wrote the first draft of the study. B.A.K. contributed to the interpretation of the data. All authors contributed to the revision of the manuscript and approved the final version for publication. S.K.F. and B.A.K. had access to all the raw data.

## Funding

This study was funded by the Northern Norway Regional Health Authority (181021).

## Conflicts of Interest

The authors declare no conflicts of interest.

## Supporting information


**Table S1A:** EQ‐5D‐5L patient‐reported dimension frequencies, counts, and percentages (*n* = 39).
**Table S1B:** EQ‐5D‐5L proxy‐reported dimension frequencies, counts, and percentages (*n* = 306).
**Table S2:** EQ‐5D‐5L scores for the general population and ICU patients stratified by sex.
**Table S3A:** Percentage (95% CI) reporting problems (score > 1) on EQ‐5D‐5L dimensions in the general population by age.
**Table S3B:** Percentage (95% CI) reporting problems (score > 1) on EQ‐5D‐5L dimensions in the intensive care population by age.
**Table S4A:** EQ‐5D‐5L frequencies (%) and descriptive statistics for the women in general population and intensive care patients (*n* = 115 per cohort).
**Table S4B:** EQ‐5D‐5L frequencies (%) and descriptive statistics for the men in general population and intensive care patients (*n* = 230 per cohort).
**Figure S1A:** Prevalence of EQ‐5D‐5L health problems (score > 1) by dimension and age group in men. Bars represent the percentage of participants reporting problems in each dimension for the intensive‐care and the norm cohorts. Error bars indicate 95% confidence intervals; *p*‐value thresholds are shown with stars (***< 0.001, **< 0.01, *< 0.05).
**Figure S1B:** Prevalence of EQ‐5D‐5L health problems (score > 1) by dimension and age group in women. Bars represent the percentage of participants reporting problems in each dimension for the intensive‐care and the norm cohorts. Error bars indicate 95% confidence intervals; *p*‐value thresholds are shown with stars (***< 0.001, **< 0.01, *< 0.05).
**Figure S2:** Association between pre‐ICU health‐related quality of life and comorbidity burden in elderly ICU patients. (A) EQ‐5D‐5L index scores and (B) EQ‐VAS scores plotted against the Comorbidity‐Polypharmacy Score. The solid lines represent linear regression trends with 95% confidence intervals (shaded areas). Spearman's rank correlation coefficients and *p*‐values are shown in each panel.

## Data Availability

The data that support the findings of this study are available on request from the corresponding author. The data are not publicly available due to privacy or ethical restrictions.
