# Supplementary material for: Impaired Pre‐Critical Illness Quality of Life in Elderly ICU Patients
Source: Acta Anaesthesiol Scand. 2026 Jul 26;70(8):e70304. doi: 10.1111/aas.70304 (PMC13401743; doi:10.1111/aas.70304)
Supplement: Supplementary file 1 — Table S1A: EQ‐5D‐5L patient‐reported dimension frequencies, counts, and percentages (n = 39). Table S1B: EQ‐5D‐5L proxy‐reported dimension frequencies, counts, and percentages (n = 306). Table S2: EQ‐5D‐5L scores for the general population and ICU patients stratified by sex. Table S3A: Percentage (95% CI) reporting problems (score > 1) on EQ‐5D‐5L dimensions in the general population by age. Table S3B: Percentage (95% CI) reporting problems (score > 1) on EQ‐5D‐5L dimensions in the intensive care population by age. Table S4A: EQ‐5D‐5L frequencies (%) and descriptive statistics for the women in general population and intensive care patients (n = 115 per cohort). Table S4B: EQ‐5D‐5L frequencies (%) and descriptive statistics for the men in general population and intensive care patients (n = 230 per cohort). Figure S1A: Prevalence of EQ‐5D‐5L health problems (score > 1) by dimension and age group in men. Bars represent the percentage of participants reporting problems in each dimension for the intensive‐care and the norm cohorts. Error bars indicate 95% confidence intervals; p‐value thresholds are shown with stars (***< 0.001, **< 0.01, *< 0.05). Figure S1B: Prevalence of EQ‐5D‐5L health problems (score > 1) by dimension and age group in women. Bars represent the percentage of participants reporting problems in each dimension for the intensive‐care and the norm cohorts. Error bars indicate 95% confidence intervals; p‐value thresholds are shown with stars (***< 0.001, **< 0.01, *< 0.05). Figure S2: Association between pre‐ICU health‐related quality of life and comorbidity burden in elderly ICU patients. (A) EQ‐5D‐5L index scores and (B) EQ‐VAS scores plotted against the Comorbidity‐Polypharmacy Score. The solid lines represent linear regression trends with 95% confidence intervals (shaded areas). Spearman's rank correlation coefficients and p‐values are shown in each panel. [file AAS-70-0-s001.docx]

**Supplementary Material**

Table S1A. EQ-5D-5L patient-reported dimension frequencies, counts, and percentages (n=39)

| EQ-5D dimension/ scores, n (%) | No problems | Slight problems | Moderate problems | Severe problems | Unable/  extreme |
| --- | --- | --- | --- | --- | --- |
| Mobility | 27 (69.2) | 7 (17.9) | 2 (5.1) | 3 (7.7) | 0 (0.0) |
| Self-care | 38 (97.4) | 1 (2.6) | 0 (0.0) | 0 (0.0) | 0 (0.0) |
| Usual activities | 25 (64.1) | 11 (28.2) | 2 (5.1) | 1 (2.6) | 0 (0.0) |
| Pain/discomfort | 26 (66.7) | 10 (25.6) | 2 (5.1) | 0 (0.0) | 1 (2.6) |
| Anxiety/depression | 35 (89.7) | 1 (2.6) | 3 (7.7) | 0 (0.0) | 0 (0.0) |

Table S1B. EQ-5D-5L proxy-reported dimension frequencies, counts, and percentages (n=306)

| EQ-5D dimension/ scores, n (%) | No problems | Slight problems | Moderate problems | Severe problems | Unable/  extreme |
| --- | --- | --- | --- | --- | --- |
| Mobility | 133 (43.5) | 76 (24.8) | 56 (18.3) | 34 (11.1) | 7 (2.3) |
| Self-care | 244 (79.7) | 29 (9.5) | 18 (5.9) | 11 (3.6) | 4 (1.3) |
| Usual activities | 177 (57.8) | 51 (16.7) | 41 (13.4) | 27 (8.8) | 10 (3.3) |
| Pain/discomfort | 75 (24.6) | 127 (41.6) | 64 (21.0) | 31 (10.2) | 8 (2.6) |
| Anxiety/depression | 159 (52.1) | 86 (28.2) | 47 (15.4) | 12 (3.9) | 1 (0.3) |

# Table S2. EQ-5D-5L scores for the general population and ICU patients stratified by sex.

|  | Female (n = 115) | | Male (n = 230) | |
| --- | --- | --- | --- | --- |
|  | Norms | ICU | Norms | ICU |
| *Dimensions* |  |  |  |  |
| Mobility | 1.0 (0.0) | 2.0 (2.0) | 1.0 (1.0) | 2.0 (2.0) |
| Self-care | 1.0 (0.0) | 1.0 (0.0) | 1.0 (0.0) | 1.0 (0.0) |
| Usual activities | 1.0 (0.0) | 1.0 (1.0) | 1.0 (1.0) | 1.0 (1.0) |
| Pain/discomfort | 2.0 (1.0) | 2.0 (1.0) | 2.0 (1.0) | 2.0 (2.0) |
| Anxiety/depression | 1.0 (0.0) | 2.0 (2.0) | 1.0 (1.0) | 1.0 (1.0) |
| EQ-5D-5L-Index  EQ VAS | 0.941 (0.131)  85.00 (15.00) | 0.845 (0.277)  68.50 (30.00) | 0.941 (0.159)  85.00 (24.50) | 0.876 (0.215)  65.00 (35.00) |
|  |  |  |  |  |

**Median (interquartile range)**. n reflect the number of participants.

Table S3A. Percentage (95% CI) reporting problems (score > 1) on EQ- 5D-5L dimensions in the general population by age.

| Variable | 65–72 years (n=140) | 73–79 years (n=143) | 80+ yrs (n=62) |
| --- | --- | --- | --- |
| Mobility | 14.3 9.4–21.0) | 30.8 (23.8–38.8) | 41.9 (30.5–54.3) |
| Self-care | 5.7 (2.9–10.9) | 8.4 (4.9–14.1) | 21.0 (12.7–32.6) |
| Usual activities | 17.9 (12.4–25.0) | 25.2 (18.8–32.9) | 37.1 (26.2–49.5) |
| Pain/discomfort | 57.9 (49.6–65.7) | 69.9 (62.0–76.8) | 71.0 (58.7–80.8) |
| Anxiety/depression | 22.9 (16.7–30.5) | 25.2 (18.8–32.9) | 25.8 (16.6–37.9) |

n reflect the number of participants.

Table S3B. Percentage (95% CI) reporting problems (score > 1) on EQ- 5D-5L dimensions in the intensive care population by age.

| Variable | 65–72 years (n=140) | 73–79 years (n=143) | 80+ yrs (n=62) |
| --- | --- | --- | --- |
| Mobility | 45.7 (37.7–54.0) | 55.9 (47.8–63.8) | 66.1 (53.7–76.7) |
| Self-care | 20.0 (14.2–27.4) | 16.1 (11.0–23.0) | 19.4 (11.4–30.9) |
| Usual activities | 39.3 (31.6–47.6) | 42.7 (34.8–50.9) | 43.5 (31.9–55.9) |
| Pain/discomfort | 70.0 (62.0–77.0) | 70.4 (62.5–77.3) | 72.6 (60.4–82.1) |
| Anxiety/depression | 46.4 (38.4–54.7) | 44.4 (36.5–52.6) | 35.5 (24.7–47.9) |

n reflect the number of participants.

Table S4A. EQ-5D-5L frequencies (%) and descriptive statistics for the women in general population and intensive care patients (n=115 per cohort)

| EQ-5D dimension/ scores, n (%) | | No problems | Slight problems | Moderate problems | Severe problems | Unable/  extreme | Sign. |
| --- | --- | --- | --- | --- | --- | --- | --- |
| General population | |  |  |  |  |  |  |
| Mobility | | 90 (78.3) | 14 (12.2) | 5 (4.3) | 5 (4.3) | 1 (0.9) |  |
| Self-care | | 100 (87.0) | 13 (11.3) | 0 (0.0) | 1 (0.9) | 1 (0.9) |  |
| Usual activities | | 94 (81.7) | 13 (11.3) | 4 (3.5) | 2 (1.7) | 2 (1.7) |  |
| Pain/discomfort | | 44 (38.3) | 53 (46.1) | 8 (7.0) | 8 (7.0) | 2 (1.7) |  |
| Anxiety/depression | | 91 (79.1) | 16 (13.9) | 7 (6.1) | 1 (0.9) | 0 (0.0) |  |
|  | |  |  |  |  |  |  |
| Intensive care | |  |  |  |  |  |  |
| Mobility | | 50 (43.5) | 26 (22.6) | 23 (20.0) | 13 (11.3) | 3 (2.6) | ** |
| Self-care | | 91 (79.1) | 13 (11.3) | 5 (4.3) | 5 (4.3) | 1 (0.9) | ns |
| Usual activities | | 64 (55.7) | 24 (20.9) | 14 (12.2) | 8 (7.0) | 5 (4.3) | ** |
| Pain/discomfort | | 27 (23.5) | 46 (40.0) | 26 (22.6) | 12 (10.4) | 4 (3.5) | ** |
| Anxiety/depression | | 56 (48.7) | 29 (25.2) | 23 (20.0) | 6 (5.2) | 1 (0.9) | ** |
|  |  | |  |  |  |  |  |

*p<0.05; **p<0.01. ns= not significant. Reported p-values for dimensions are adjusted for multiple comparisons using the Benjamini-Hochberg procedure.

Table S4B. EQ-5D-5L frequencies (%) and descriptive statistics for the men in general population and intensive care patients (n=230 per cohort)

| EQ-5D dimension/ scores, n (%) | | No problems | Slight problems | Moderate problems | Severe problems | Unable/  extreme | Sign. |
| --- | --- | --- | --- | --- | --- | --- | --- |
| General population | |  |  |  |  |  |  |
| Mobility | | 165 (71.7) | 41 (17.8) | 14 (6.1) | 9 (3.9) | 1 (0.4) |  |
| Self-care | | 212 (92.2) | 14 (6.1) | 3 (1.3) | 1 (0.4) | 0 (0.0) |  |
| Usual activities | | 167 (72.6) | 42 (18.3) | 12 (5.2) | 7 (3.0) | 2 (0.9) |  |
| Pain/discomfort | | 76 (33.0) | 105 (45.7) | 31 (13.5) | 15 (6.5) | 3 (1.3) |  |
| Anxiety/depression | | 170 (73.9) | 44 (19.1) | 15 (6.5) | 1 (0.4) | 0 (0.0) |  |
|  | |  |  |  |  |  |  |
| Intensive care | |  |  |  |  |  |  |
| Mobility | | 110 (47.8) | 57 (24.8) | 35 (15.2) | 24 (10.4) | 4 (1.7) | ** |
| Self-care | | 191 (83.0) | 17 (7.4) | 13 (5.7) | 6 (2.6) | 3 (1.3) | ** |
| Usual activities | | 138 (60.0) | 38 (16.5) | 29 (12.6) | 20 (8.7) | 5 (2.2) | ** |
| Pain/discomfort | | 74 (32.3) | 91 (39.7) | 40 (17.5) | 19 (8.3) | 5 (2.2) | ns |
| Anxiety/depression | | 138 (60.3) | 58 (25.3) | 27 (11.8) | 6 (2.6) | 0 (0.0) | ** |
|  |  | |  |  |  |  |  |

*p<0.05; **p<0.01. ns= not significant. Reported p-values for dimensions are adjusted for multiple comparisons using the Benjamini-Hochberg procedure.


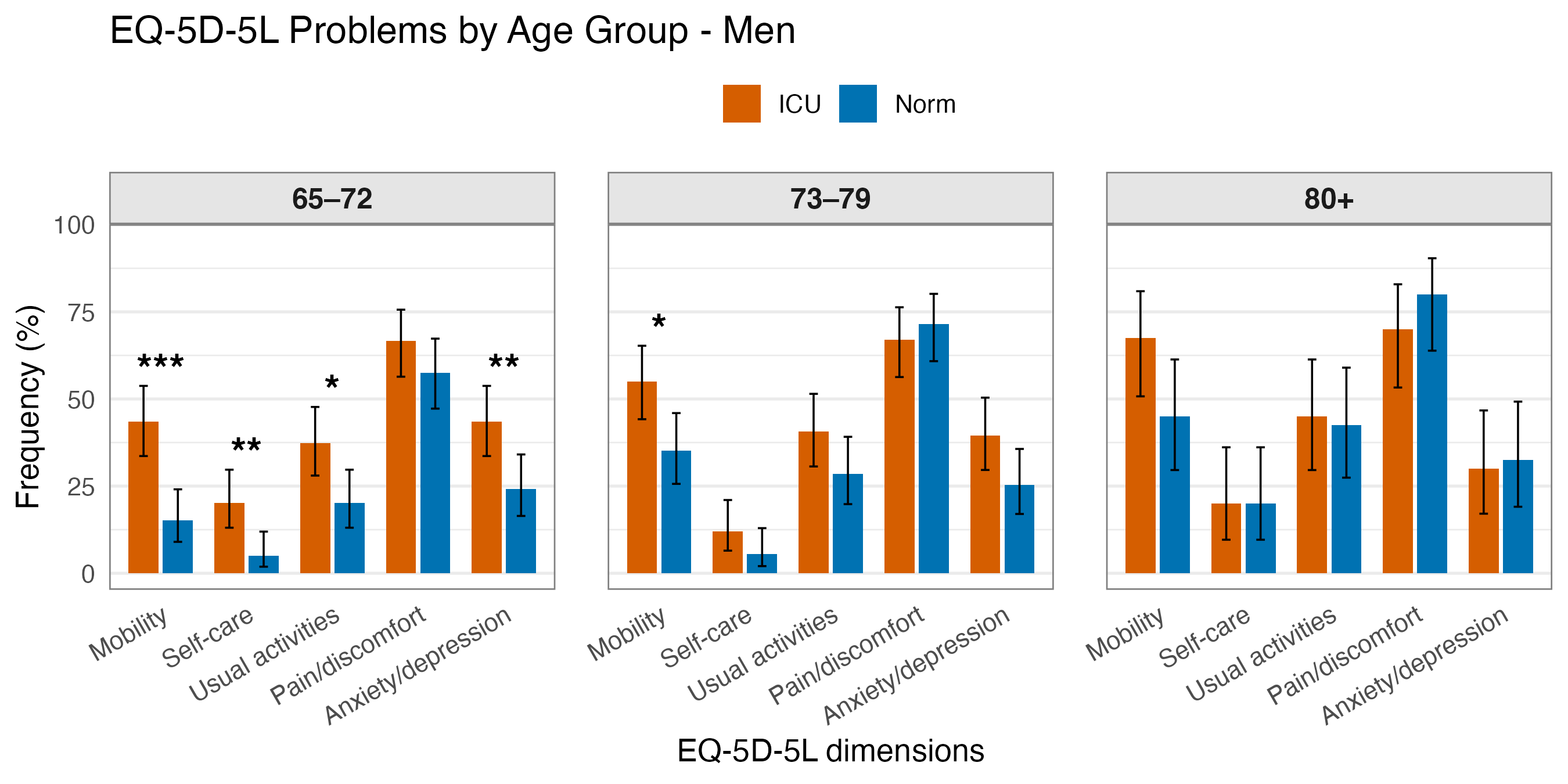


Figure S1A. Prevalence of EQ-5D-5L health problems (score > 1) by dimension and age group in men. Bars represent the percentage of participants reporting problems in each dimension for the intensive-care and the norm cohorts. Error bars indicate 95% confidence intervals; *p*-value thresholds are shown with stars (*** < 0.001, ** < 0.01, * < 0.05).


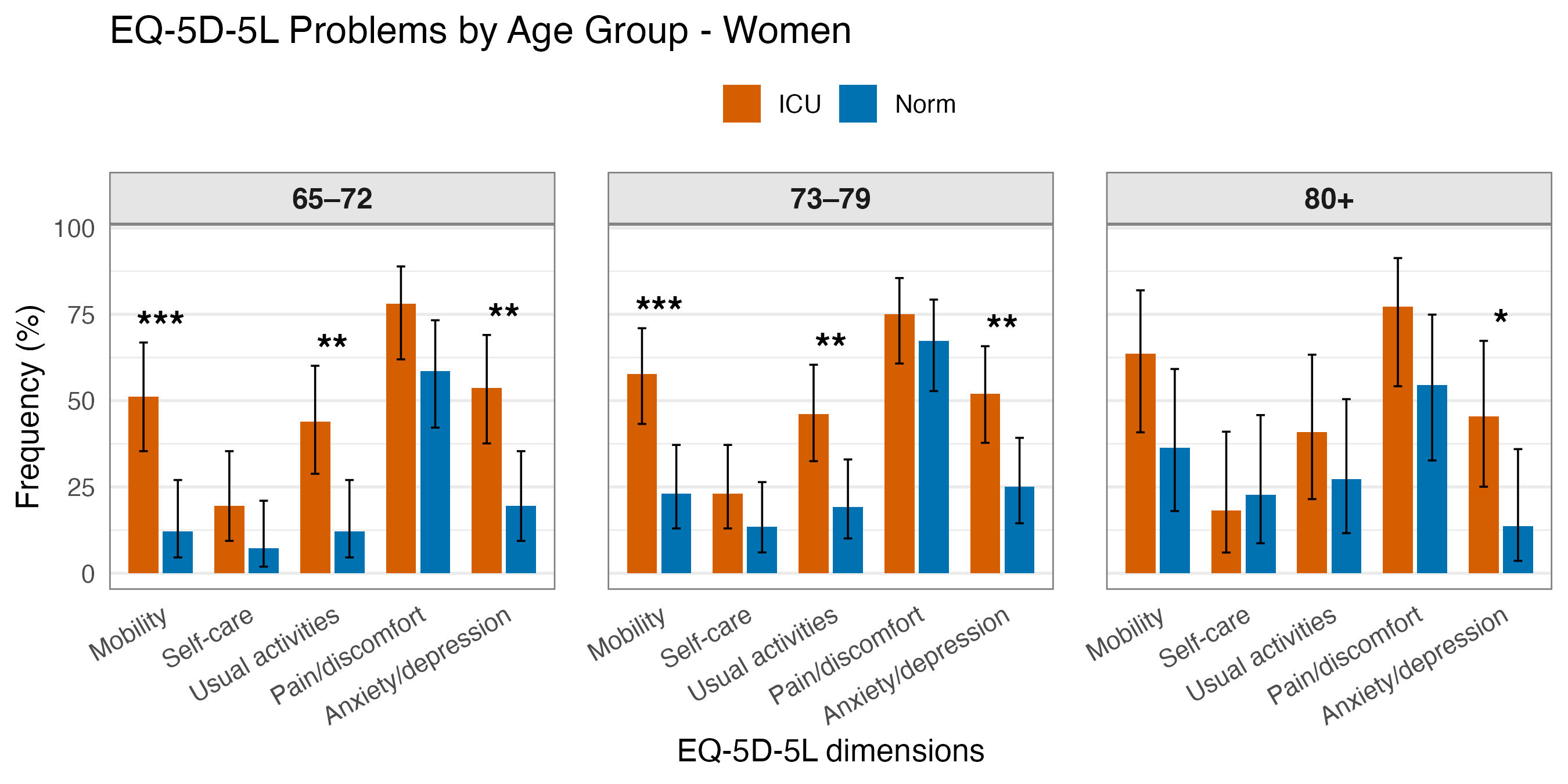


Figure S1B. Prevalence of EQ-5D-5L health problems (score > 1) by dimension and age group. in women. Bars represent the percentage of participants reporting problems in each dimension for the intensive-care and the norm cohorts. Error bars indicate 95% confidence intervals; *p*-value thresholds are shown with stars (*** < 0.001, ** < 0.01, * < 0.05).

Figure S2. Association between pre-ICU health-related quality of life and comorbidity burden in elderly ICU patients.  (A)  EQ-5D-5L index scores and (B) EQ-VAS scores plotted against the Comorbidity-Polypharmacy Score. The solid lines represent linear regression trends with 95% confidence intervals (shaded areas). Spearman’s rank correlation coefficients and p-values are shown in each panel.
